# A Meta-Analysis to Determine Strength Training Related Dose-Response Relationships for Lower-Limb Muscle Power Development in Young Athletes

**DOI:** 10.3389/fphys.2018.01155

**Published:** 2018-08-22

**Authors:** Maamer Slimani, Armin Paravlic, Urs Granacher

**Affiliations:** ^1^Centre National de la Medecine et des Sciences Des Sports, Tunis, Tunisia; ^2^Scientific Research Center Koper, Institute for Kinesiology Research, Koper, Slovenia; ^3^Division of Training and Movement Sciences, Research Focus Cognition Sciences, University of Potsdam, Potsdam, Germany

**Keywords:** resistance training, muscle fitness, youth, meta-analysis, jump performance

## Abstract

It is well-documented that strength training (ST) improves measures of muscle strength in young athletes. Less is known on transfer effects of ST on proxies of muscle power and the underlying dose-response relationships. The objectives of this meta-analysis were to quantify the effects of ST on lower limb muscle power in young athletes and to provide dose-response relationships for ST modalities such as frequency, intensity, and volume. A systematic literature search of electronic databases identified 895 records. Studies were eligible for inclusion if (i) healthy trained children (girls aged 6–11 y, boys aged 6–13 y) or adolescents (girls aged 12–18 y, boys aged 14–18 y) were examined, (ii) ST was compared with an active control, and (iii) at least one proxy of muscle power [squat jump (SJ) and countermovement jump height (CMJ)] was reported. Weighted mean standardized mean differences (SMDwm) between subjects were calculated. Based on the findings from 15 statistically aggregated studies, ST produced significant but small effects on CMJ height (SMDwm = 0.65; 95% CI 0.34–0.96) and moderate effects on SJ height (SMDwm = 0.80; 95% CI 0.23–1.37). The sub-analyses revealed that the moderating variable expertise level (CMJ height: *p* = 0.06; SJ height: N/A) did not significantly influence ST-related effects on proxies of muscle power. “Age” and “sex” moderated ST effects on SJ (*p* = 0.005) and CMJ height (*p* = 0.03), respectively. With regard to the dose-response relationships, findings from the meta-regression showed that none of the included training modalities predicted ST effects on CMJ height. For SJ height, the meta-regression indicated that the training modality “training duration” significantly predicted the observed gains (*p* = 0.02), with longer training durations (>8 weeks) showing larger improvements. This meta-analysis clearly proved the general effectiveness of ST on lower-limb muscle power in young athletes, irrespective of the moderating variables. Dose-response analyses revealed that longer training durations (>8 weeks) are more effective to improve SJ height. No such training modalities were found for CMJ height. Thus, there appear to be other training modalities besides the ones that were included in our analyses that may have an effect on SJ and particularly CMJ height. ST monitoring through rating of perceived exertion, movement velocity or force-velocity profile could be promising monitoring tools for lower-limb muscle power development in young athletes.

## Introduction

Coaches' and fitness professionals' daily task is to enhance performance of their athletes using effective and efficient training regimes. High levels of muscle power represent important performance determinants in several sports (e.g., athletics, combat sports, rugby) and are related to success in sport competition (James et al., 2016; Slimani and Nikolaidis, 2017). Accordingly, Granacher et al. (2016) postulated that muscle power should be systematically developed during daily strength and conditioning routines in athletes. Athletes' performance in muscle power can be estimated using different tests. Vertical jump tests represent easy-to-administer, frequently used, and reliable tests for the assessment of muscle power. Markovic et al. (2004) reported that the countermovement jump (CMJ) and squat jump (SJ) tests are well-suited since they afford complex motor coordination between upper- and lower-body segments and because performance-related measures like jump height are highly associated with power measures (Kons et al., 2018).

The development of muscle power is not only important in elite adult athletes but also in child and particularly in adolescent athletes. Lloyd and Oliver (2012) provided evidence in their youth physical development model that muscle power should be developed during all periods of maturation [i.e., pre, around, post peak-height-velocity (PHV)]. Youth with insufficient levels of physical fitness (e.g., muscular power and strength) who do not become proficient movers early in life will be less likely to participate in diverse physical activities as adults (Robinson et al., 2015). Thus, it is important to improve muscle power early in life to avoid neuromuscular deficiencies and adverse health events later in life (Bergeron et al., 2015). For young athletes, there is evidence that adequately designed strength and conditioning programs have the potential to stimulate motor/athletic development and help prevent acute and overuse injuries (Faigenbaum et al., 2016; Lloyd et al., 2016).

Granacher et al. (2016) followed up on the youth physical development model of Lloyd and Oliver and introduced a conceptual model on the implementation of strength training (ST) during the different stages of long-term athlete development. In accordance with Lloyd and Oliver (2012), Granacher et al. (2016) recommended to implement maturation and expertise related types of ST during all stages of long-term athlete development.

However and somewhat in contrast to the aforementioned models, previous studies reported either small or controversial effects of ST on proxies of muscle power (e.g., CMJ height) in children and adolescents (Weltman et al., 1986; Lillegard et al., 1997; Faigenbaum et al., 2002; Christou et al., 2006; Granacher et al., 2016). These controversial findings from original studies were confirmed by systematic reviews and meta-analysis that examined the effects of strength and/or power training on proxies of muscle power in trained and untrained children and adolescents (Behm et al., 2008, 2017; Lesinski et al., 2016). For instance, Lesinski et al. (2016) computed effects and dose-response relationships of ST for measures of muscle strength in young athletes. These authors revealed moderate ST-related effects on muscle strength and vertical jump performances and small effects for linear sprint, agility, and sport-specific performances. In another meta-analysis, Behm et al. (2017) aggregated findings from 107 studies and reported the effects of strength vs. power training on measures of strength, power, and speed in youth. These authors postulated that power training was more effective than ST for improving jump performances in children and adolescents. Of note, ST was more effective than power training to improve strength and sprint performances (Behm et al., 2017). These inconsistent results have been attributed to several factors, including differences in the applied testing methods, expertise level, age, maturational status (Behm et al., 2017), and training modalities (Behringer et al., 2011). Based on their findings, Behm et al. (2017) recommended to conduct ST during the early stages of maturation and/or long-term athlete development and power training during the later stages. The sequencing of ST prior to power training raises the question whether ST-related adaptations translate to proxies of muscle power and if there are effective dose-response relations. This research topic could be addressed using a meta-analytical approach to detect transfer effects of ST on proxies of muscle power and to identify effective dose-response relationships in youth.

Previous studies computed dose-response relationships following ST for measures of muscle strength but not muscle power in adolescents. To the best of the authors' knowledge, there is no published meta-analysis available that examined dose-response relationships following ST on proxies of lower limbs muscle power, such as CMJ and SJ height in young athletes. Thus, in an effort to complement the findings of Lesinski et al. (2016) and Behm et al. (2017), we conducted a meta-analysis and aimed at examining the effects of ST on proxies of lower limb muscle power in healthy child and adolescent athletes. In addition, we quantified ST specific dose-response relationships for proxies of muscle power according to the training modalities intensity, frequency, and volume.

## Materials and methods

### Search strategy

**Figure 1:**
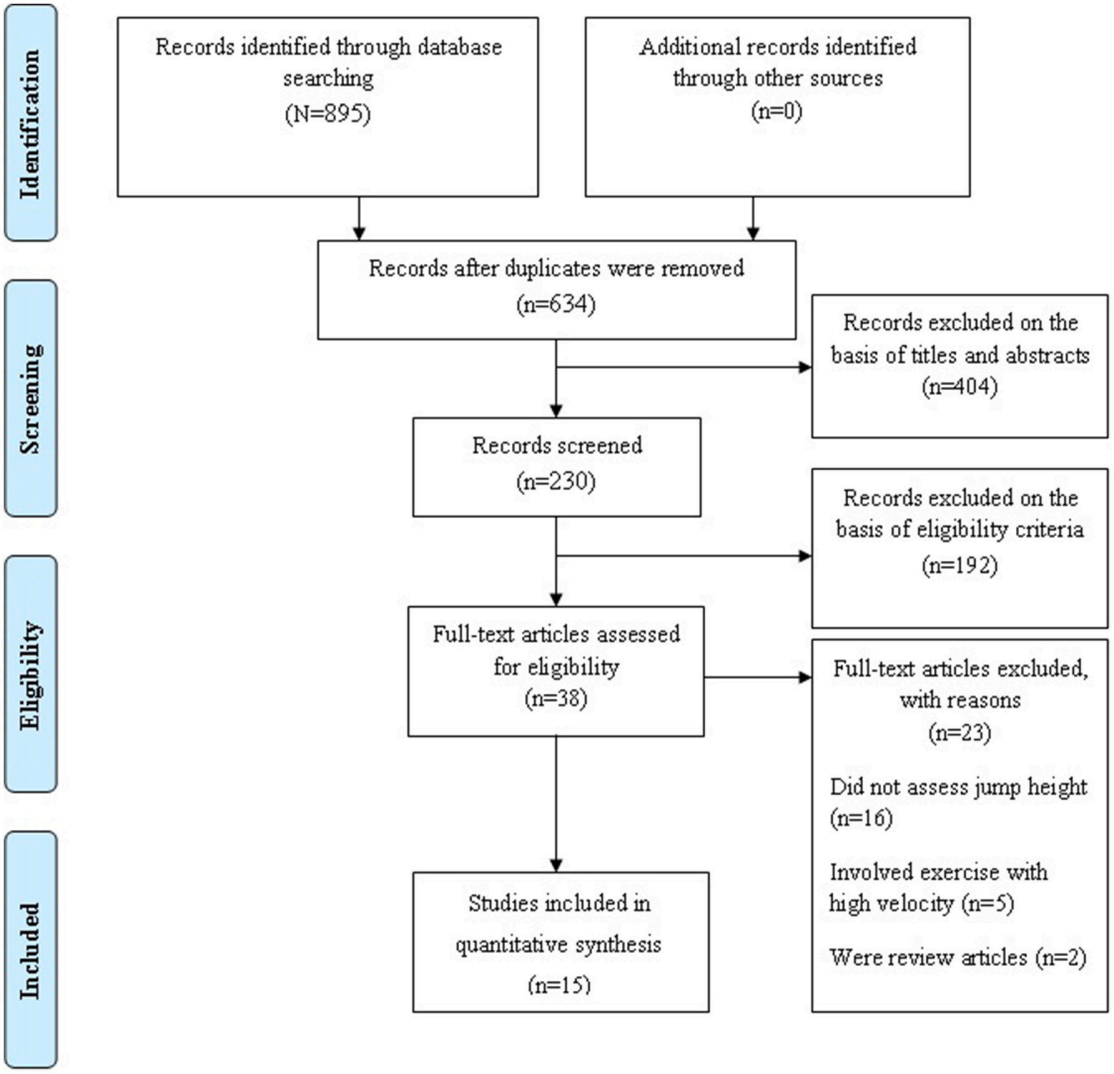
Preferred Reporting Items for Systematic Reviews and Meta-analysis (PRISMA) flow-chart.

This meta-analysis was conducted in accordance with the Preferred Reporting Items for Systematic Reviews and Meta-Analysis (PRISMA) guidelines (Figure 1, Moher et al., 2009). A systematic literature search was performed for randomized controlled trials (RCTs) that studied the effects of ST on CMJ and SJ height in healthy child and adolescent athletes. In accordance with Behm et al. (2017), ST has been defined as any isometric, traditional free weight or machine-based (i.e., isoinertial or isokinetic) type of resistance exercise that was performed at slow or moderate movement velocity. In contrast, power training refers to a type of exercise that requires high movement speed and explosive muscle actions. Accordingly, plyometric training studies were categorized as power training and therefore not included in this systematic review and meta-analysis (Behm et al., 2017). Studies were obtained through systematic manual and electronic searches (up to March 1st, 2018) in electronic databases (i.e., Google Scholar, MEDLINE/PubMed, SpringerLink, ScienceDirect Journals, Taylor & Francis Online—Journals). Electronic databases were searched using the following search syntax with keywords and/or MeSH terms: [(“strength training” OR “resistance training” OR “weight training” OR “weight-bearing exercise”) AND (child OR children OR adolescent OR youth OR young OR puberty OR pubertal OR prepubertal OR kid OR teen OR girl OR boy) AND (“squat jump” OR “countermovement jump”)]. Moreover, we performed manual searches of relevant journals and reference lists obtained from published articles. The present meta-analysis included studies published in journals that reported original research data from healthy children and adolescents.

### Risk of bias

According to the Cochrane Collaboration guidelines (Higgins and Green, 2011), two authors independently assessed the methodological quality and risk of bias via visual interpretation of funnel plots.

### Inclusion and exclusion criteria

Studies were included in this review if they met all the following Population/Intervention /Comparison/Outcome(s) (PICOS) criteria:

1. Population: Studies recruiting healthy recreationally trained males and females (i.e., physical education students) and/or trained (i.e., high-level, professional, elite, national) child and adolescent athletes as participants;
2. Intervention or exposure: Studies examining the effects of ST on proxies of muscle power (i.e., SJ and CMJ height);
3. Comparator: Studies involving an active control group against which an intervention was compared;
4. Outcome(s): Studies were identified that assessed SJ and CMJ height as proxies of muscle power. In addition, we examined how moderating variables like training duration (weeks), training frequency (sessions/week), training intensity (% of one-repetition maximum [1RM]), number of exercises per session, number of sets per exercise and number of repetitions per set, influenced ST-related performance enhancements;
5. Study design: Original research in the form of RCTs; Studies were excluded if […]:
  - they were reviews, comments, opinion papers and commentaries, interviews, letters to the editor, editorials, posters, conference papers, abstracts, book chapters, and books. However, published review articles were examined to avoid that we missed potentially relevant articles;
  - they did not follow an experimental approach and reported valid and reliable measurements;
  - they did not include sufficient data to calculate standardized mean differences.

### Coding of studies

Two authors independently extracted data using a structured form. Because of the high number of potential variables that may affect training effectiveness, independent variables were grouped into the following categories according to Raymond et al. (2013): (i) type of intervention: strength group vs. active control; (ii) age (children: girls aged 6–11 y, boys aged 6–13 y; adolescents: girls aged 12–18 y, boys aged 14–18 y), expertise level (recreationally trained vs. trained), and sex (males vs. combined males and females); and (iii) program modalities (training duration in weeks [< 8 vs. ≥8 weeks], weekly training frequency [2 vs. 3 sessions per week], number of sets per exercise [1 vs. 3 vs. 4 vs. 5], number of repetitions per set [4 vs. 5 vs. 6 vs. 7 vs. 10 vs. 11 vs. 12], number of exercises per session [1 vs. 2 vs. 3 vs. 4 vs. 5 vs. 6 vs. 7], and training intensity [high-intensity: ≥ 70% of the 1RM; moderate-intensity: 51% ≥ 1RM ≤ 69%; and low-intensity: ≤ 50% 1RM]).

### Data extraction

The main study characteristics (i.e., cohort, age, intervention program, training variables, relevant outcomes) were extracted in an Excel template/spreadsheet.

### Statistical analyses

In this meta-analysis, data were extracted from the included studies using a standardized documentation form. Weighted mean standardized mean differences between subjects (SMDwm) with 95% confidence intervals (95% CI) were calculated for the identified studies. Meta-analyses were computed using the program Comprehensive Meta-Analysis, version 2 (Borenstein et al., 2005). Statistical heterogeneity was assessed using *Q* and *I*^2^ statistics. The *I*^2^ measure of inconsistency was used to examine between-study variability. Values of 25, 50, and 75% represent low, moderate, and high statistical heterogeneity (Higgins et al., 2003). Due to study heterogeneity, we decided to apply a random-effects model in all comparisons. Potential publication bias was visually inspected with a funnel plot, looking at asymmetry of the graph. In addition, meta-regression analyses (method of moments) were applied to compute possible predictors that may have influenced training-related effects (e.g., training duration, weekly training frequency, number of exercises, number of sets per training and number of repetitions per sets). According to Rhea et al. (2003), SMDwm magnitudes were classified as trivial (<0.35), small (0.35–0.80), moderate (0.80–1.50), or large (>1.5). The significance level was set at *p* < 0.05.

## Results

### Study characteristics

The applied search strategy yielded a preliminary number of 895 studies that was eligible for inclusion in this meta-analysis. After the screening of titles and abstracts, 230 papers remained. Full texts of 38 articles were retrieved and assessed using inclusion and exclusion criteria. After a careful review of the full texts, 23 articles were excluded and the remaining 15 articles were included in this meta-analysis. A flow chart of the systematic search process is illustrated in Figure 1. Details of all included studies are depicted in Table 1.

**Table 1:** Descriptive analysis of the included studies.

	**Group**	**Sex**	**N**	**Age**	**Level**	**Weeks**	**Sessions per week**	**% 1RM**	**Number of exercises**	**Number of sets**	**Number of reps**	**SJ (cm)**		**CMJ (cm)**	
												**Pre**	**Post**	**Pre**	**Post**
Channell and Barfield, 2008	SG	M	11	Adolescents	T	8	3	60–100	3	4	11			57.5 ± 7.2	60.1 ± 3.9
	CG	M	6	Adolescents	T	8								59.1 ± 9.1	57.4 ± 7.7
Chelly et al., 2009	SG	M	11	Adolescents	T	8	2	70–90	1	4	4	31.5 ± 4	34.6 ± 3	33.8 ± 4	36.3 ± 3
	CG	M	11	Adolescents	T	8						30.8 ± 3.6	31.4 ± 3.5	33.8 ± 3.7	34.5 ± 4.2
Christou et al., 2006	SG	M	9	Adolescents	T	8	2	55–80	10	3	12	24.9 ± 1.4	28.1 ± 1.4	29 ± 1.6	32.9 ± 1.4
	CG	M	8	Adolescents	T	8						25 ± 2	26.5 ± 1.8	29 ± 2	30.6 ± 1.4
Christou et al., 2006	SG	M	9	Adolescents	T	16	2	55–80	10	3	12	24.9 ± 1.4	32.4 ± 1.6	29 ± 1.6	35.7 ± 1.4
	CG	M	8	Adolescents	T	16						25 ± 2	27 ± 2.1	29 ± 2	31.2 ± 1.5
de Hoyo et al., 2016	SG	M	18	Adolescents	T	10	1–2		2	4	6			35.7 ± 4.1	38.3 ± 4.2
	CG	M	15	Adolescents	T	10	1-2							36.8 ± 3.4	36.2 ± 3.2
Enoksen et al., 2013	SG	M	9	Adolescents	T	10	2	70–95	3	3	7			33.7 ± 6.3	36.4 ± 6.0
	CG	M	9	Adolescents	T	10								34.4 ± 3.4	36.3 ± 3.3
Faigenbaum et al., 2002	SG	MF	20	Children	RT	6	2	10–15 RM	12	1	12			22.8 ± 3.9	24.9 ± 4.5
	CG	MF	13	Children	RT	6								21.6 ± 2.5	22.3 ± 2.2
Gorostiaga et al., 1999	SG	M	9	Adolescents	T	6	2	40–90	5	4		32.2 ± 3.2	33.3 ±3.3	34.1 ± 3.1	35.2 ± 3.6
	CG	M	4	Adolescents	T	6						27 ± 4.0	28 ±3.2	32 ± 25.5	32.8 ± 24
Granacher et al., 2011	SG	MF	17	Children	T	10	2	70–80	7	3	11			21.5 ± 2.6	22.2 ± 2.7
	CG	MF	15	Children	T	10								20.8 ± 4.0	21.3 ± 4.6
Kotzamanidis et al., 2005	SG	M	11	Adolescents	T	9	2	8,6,3 RM	NR	4	6	25.7 ± 3.1	26.1 ± 3.4	27.2 ± 3.4	27.4 ± 3.3
	CG	M	12	Adolescents	T							25.8 ± 2.4	26.0 ± 2.5	28.3 ± 2.7	28.2 ± 2.8
Lloyd et al., 2016 (Pre-PHV)	SG	M	10	Children	T	6	2	10 RM	4	3	10	22.3 ± 4.9	24.8 ± 4.6		
	CG	M	10	Children	T							23.4 ± 4.6	23.5 ± 4.2		
Lloyd et al., 2016 (Post-PHV)	SG	M	10	Adolescents	T	6	2	10 RM	4	3	10	32.4 ± 5.0	34.6 ± 5.1		
	CG	M	10	Adolescents	T							34.2 ± 4.6	34.2 ± 4.6		
Moraes et al., 2013	SG	M	14	Adolescents	RT	4	3	10–12 RM	9	3	11			29.4 ± 6.0	30.1 ± 6.2
	CG	M	10	Adolescents	RT	4								33.5 ± 12	33.3 ± 11.7
Moraes et al., 2013	SG	M	14	Adolescents	RT	8	3	10–12 RM	9	3	11			29.4 ± 6.0	30.6 ± 5.6
	CG	M	10	Adolescents	RT	8								33.5 ± 12	32.9 ± 11.5
Moraes et al., 2013	SG	M	14	Adolescents	RT	12	3	10–12 RM	9	3	11			29.4 ± 6.0	30.8 ± 6.0
	CG	M	10	Adolescents	RT	12								33.5 ± 12	33 ± 11.5
Ronnestad et al., 2008	SG	M	10	Adolescents	T	7	2		2	4	5	29 ± 0.9	31 ± 1.7	32.3 ± 0.8	33.9 ± 0.6
	CG	M	10	Adolescents	T	7						30.3 ± 1.2	29.2 ± 1.1	36 ± 0.9	35.7 ± 1.4
Santos and Janeira, 2012	SG	M	15	Adolescents	T	10	3	10 RM	6	3	11	24.8 ± 3.3	27.9 ± 4.0	33.3 ± 4.3	36.6 ± 4.2
	CG	M	10	Adolescents	T	10						22.7 ± 4.3	20.7 ± 3.9	30.7 ± 5.1	28.4 ± 4.0
Sarabia et al., 2015	SG	M	11	Adolescents	T	11	2		2	5		28.4 ± 3.6	31.1 ± 2.2	31.1 ± 3.5	32.4 ± 2.3
	CG	M	9	Adolescents	T	11						31.7 ± 4.6	33.2 ± 3.5	33.8 ± 3.5	33.5 ± 4.4
Weltman et al., 1986	SG	M	16	Children	T	14	3		10					21.1 ± 4.8	23.3 ± 3.4
	CG	M	10	Children	T	14								22.7 ± 3.9	22.0 ± 2.5

*CG, control group; PHV, peak height velocity; M, male; MF, male and female; NR, not reported: Reps, repetitions; SG, strength group; T, trained; RT, recreationally trained*.

**Figure 2:**
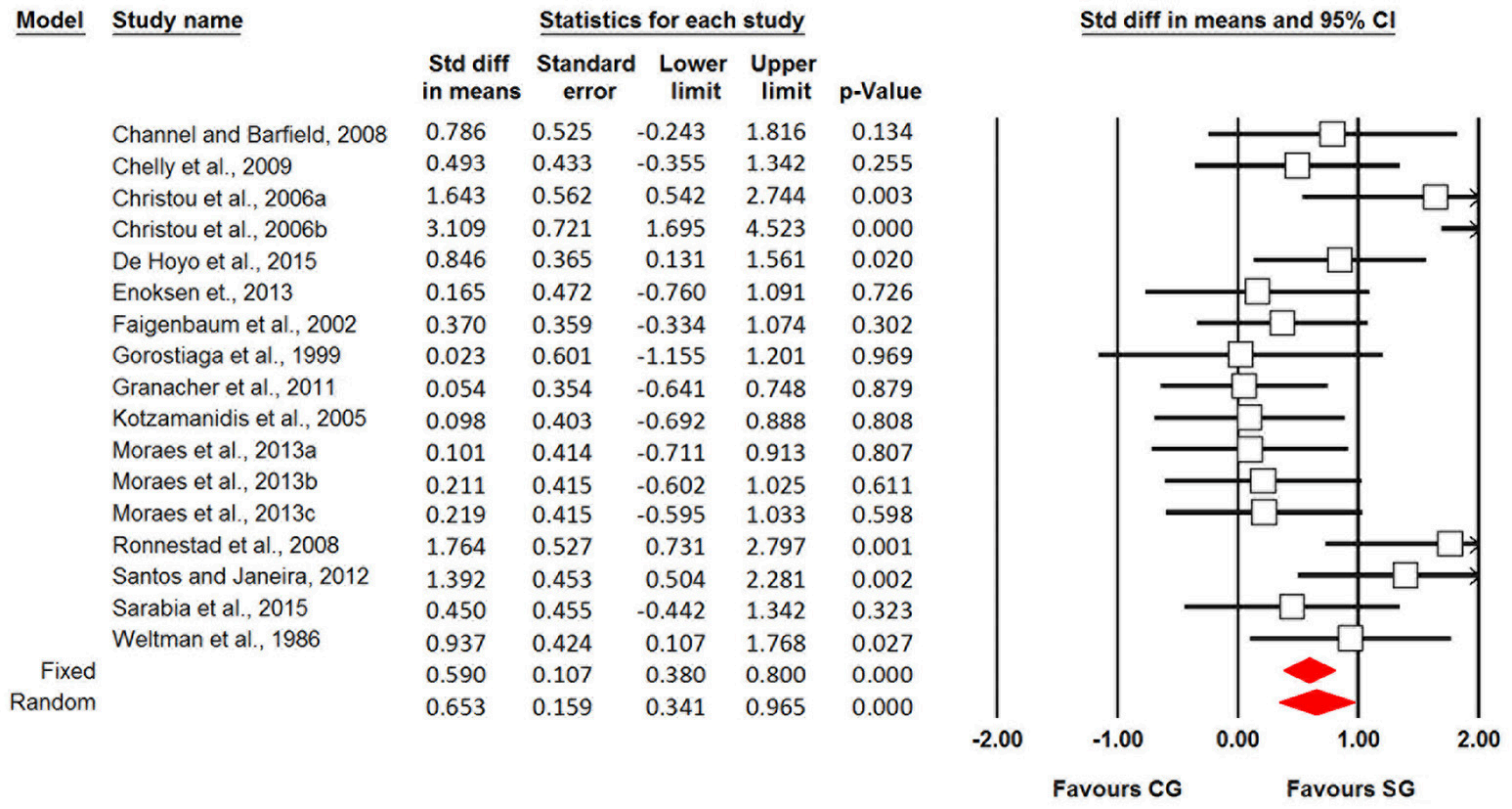
Forest plot of the standardized mean differences of the changes in countermovement jump height following strength training in young trained individuals.

**Figure 3:**
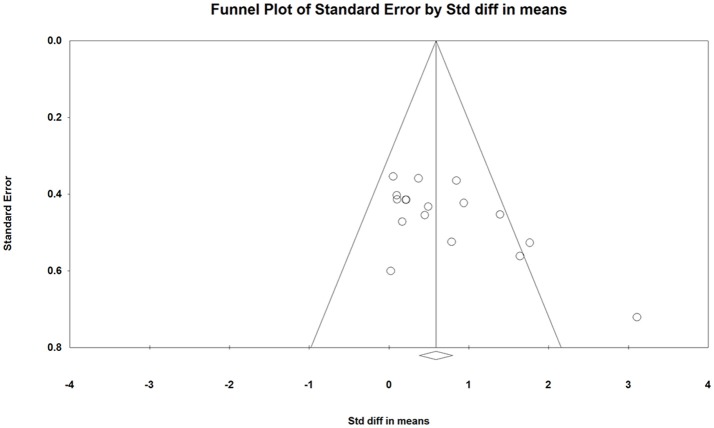
Funnel plot of the standard differences in means vs. standard error for countermovement jump height; the aggregated standard difference in means is the random effects mean effect size weighted by degrees of freedom.

Fourteen studies (17 effect sizes) examined the effects of ST on measures of CMJ height in young athletes. Our analyses revealed small ST-related effects (SMDwm = 0.65; 95% CI 0.34–0.96) for CMJ height, with moderate heterogeneity (*I*^2^ = 53.11%) (Figures 2, 3).

**Figure 4:**
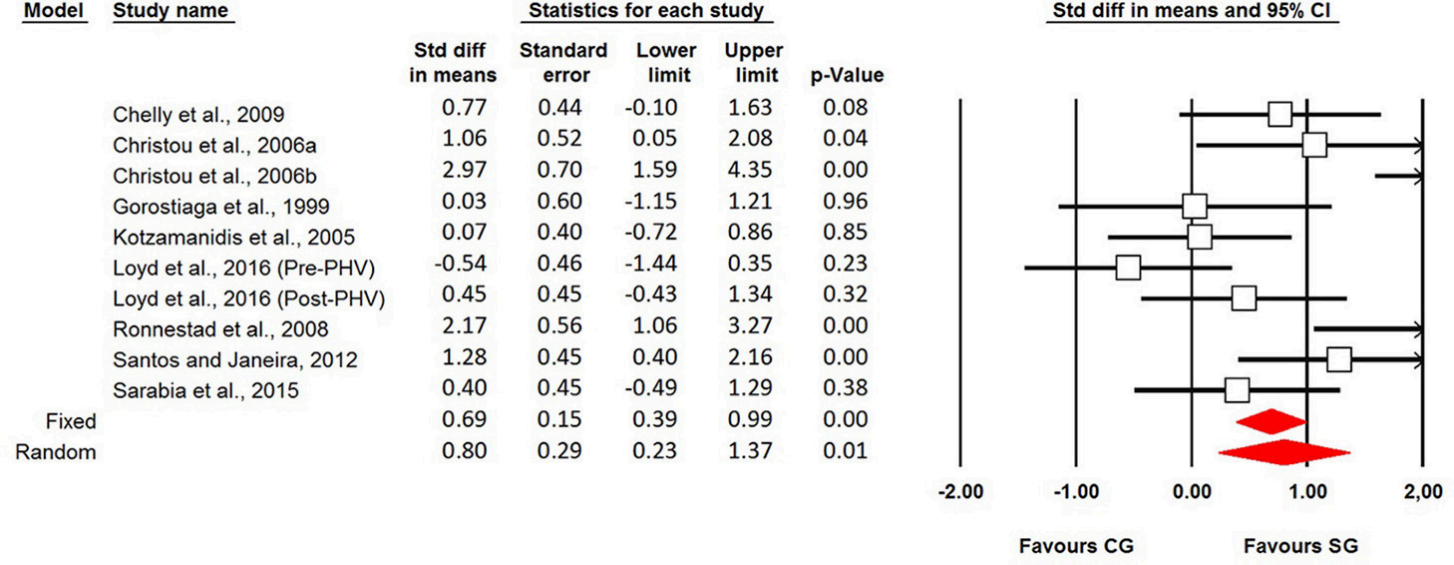
Forest plot of the standardized mean differences of the changes in squat jump height following strength training in young trained individuals.

**Figure 5:**
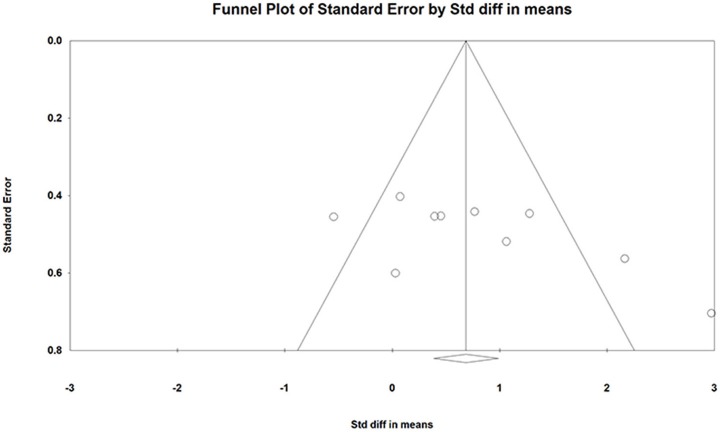
Funnel plot of the standard difference in means vs. standard error for squat jump height; the aggregated standard difference in means is the random effects mean effect size weighted by degrees of freedom.

Eight studies (9 effect sizes) were identified that reported moderate effects of ST on SJ height in young athletes with a mean SMDwm of 0.80 (95% CI 0.23–1.37; *I*^2^ = 71.19%). Moderate heterogeneity was observed (Figures 4, 5).

### Influence of different moderating variables on strength training related effects

#### Age

Subgroup analyses revealed that the moderating variable “age” (children vs. adolescents) significantly influenced ST-related changes in SJ height (*Q* = 7.76, *p* = 0.005) but not in CMJ height (*Q* = 0.74, *p* = 0.39) (Tables 2, 3). Of note, adolescents showed slightly greater gains in CMJ height (SMDwm = 0.69; 95% CI 0.29–1.08; *p* = 0.001; *df* = 11; *I*^2^ = 55.94%) compared with children (SMDwm = 0.41; 95% CI −0.0731 to 0.891; *p* = 0.09; *df* = 2; *I*^2^ = 22.22%). However, the between-group difference did not reach the level of significance. Moreover, adolescents experienced considerably greater gains in SJ height (SMDwm = 0.95; 95% CI 0.40–1.50; *p* = 0.001; *df* = 7; *I*^2^ = 65.22%) compared with children (SMDwm = −0.54; 95% CI −1.44 to 0.35; *p* = 0.23; *df* = 0; *I*^2^ = 0%).

**Table 2:** Effects of strength training on CMJ height considering different moderating variables.

**Independent variables**	**SMD**	**SE**	**95 % CI**	***p***	***I^2^*(%)**	***df***	***Q-*value and *(p)* between groups**
**SEX**							
Males	0.79	0.19	0.42–1.15	**<0.001**	54.74^**^	13	**4.56 (0.033)**
Males and females	0.18	0.21	−0.24–0.60	0.404	0.0	2	
**AGE OF ATHLETES**							
Adolescents	0.69	0.20	0.29–1.08	**0.001**	55.94^**^	11	
Children	0.41	0.25	−0.07–0.89	0.095	22.22.	2	0.74 (0.390)
**EXPERTISE LEVELS OF ATHLETE**							
Trained	0.81	0.22	0.38–1.253	**<0.001**	60.23^**^	10	3.31 (0.069)
recreationally trained	0.36	0.18	0.01–0.72	0.044	0.0	4	
**TRAINING DURATION**							
≤ 8 weeks	0.62	0.22	0.19–1.05	**0.004**	42.36	7	
> 8 weeks	0.69	0.24	0.22–1.16	**0.004**	63.58^*^	8	0.05 (0.822)
**WEEKLY TRAINING FREQUENCY**							
2 per week	0.71	0.23	0.27–1.16	**0.002**	43.16^*^	10	0.18 (0.670)
3 per week	0.58	0.21	0.17–0.99	**0.006**	28.10	5	
**INTENSITY**							
Low to high	0.02	0.60	−1.15–1.20	0.969	0.0	0	3.28 (0.350)
Moderate	0.37	0.36	−0.33–1.07	0.302	0.0	0	
Moderate to high	1.19	0.43	0.35–2.03	**0.005**	19.41	1	
High	0.52	0.24	0.05–0.99	**0.030**	62.96^**^	8	
**NUMBER OF SETS PER EXERCISE**							
1	0.37	0.36	−0.33–1.07	0.302	0.0	0	
3	0.74	0.31	0.14–1.34	**0.016**	71.91	7	
4	0.66	0.24	0.19–1.13	**0.006**	36.85	5	
5	0.45	0.46	−0.44–1.34	0.323	0.0	0	0.78 (0.853)
**NUMBER OF REPETITIONS PER SET**							
4	0.49	0.43	−0.36–1.34	0.255	0.00	0	
5	1.76	0.53	0.73–2.80	**0.001**	0.00	0	
6	0.49	0.37	−0.24–1.22	0.188	47.19	1	
7	0.17	0.47	−0.76–1.09	**0.021**	0.00	0	
11	0.1	0.21	0.00–0.82	**0.049**	30.55	5	
12	1.61	0.78	0.08–3.14	**0.039**	84.39	2	8.31 (0.140)

*CI, confidence interval; I^2^, index of heterogeneity; df, degrees of freedom; SD, standard deviation; SMD, standardized mean differences, p significance level*,

* p < 0.05;

** *p < 0.01. Bold values indicate statistically significant values*.

**Table 3:** Effects of strength training on SJ height considering different moderating variables.

**Independent variables**	**SMD**	**SE**	**95 % CI**	***P***	***I*^2^ (%)**	***df***	***Q-*value and *(p)* between groups**
**SEX**							
Males	0.89	0.32	0.27–1.51	**0.005**	71.97^**^	8	2.55 (0.111)
Males and females	0.07	0.40	−0.72–0.86	0.855	0.0	0	
**AGE OF ATHLETES**							
Adolescents	0.95	0.28	0.40–1.50	**0.001**	65.22^**^	7	
Children	−0.54	0.46	−1.44–0.35	0.232	0.0	0	7.76 (**0.005**)
**TRAINING DURATION**							
≤ 8 weeks	0.64	0.36	−0.08–1.35	0.082	68.71^*^	5	
> 8 weeks	1.07	0.54	0.02–2.12	**0.046**	79.60^**^	3	0.45 (0.501)
**WEEKLY TRAINING FREQUENCY**							
2 per week	0.74	0.32	0.12–1.37	**0.019**	72.64^**^	8	0.96 (0.328)
3 per week	1.28	0.45	0.40–2.16	**0.004**	0.0	0	
**INTENSITY**							
Moderate	1.28	0.45	0.40–2.16	**0.004**	0.0	0	1.02 (0.601)
Moderate to high	1.06	0.52	0.05–2.08	**0.041**	0.0	0	
High	0.64	0.46	−0.27–1.55	0.169	78.93^**^	4	
**NUMBER OF SETS PER EXERCISE**							
3	0.97	0.51	−0.02–1.96	0.056	80.22	4	
4	0.73	0.46	−0.17–1.64	0.110	71.19	3	0.73
5	0.40	0.45	−0.49–1.29	0.383	0.0	0	(0.694)
**NUMBER OF REPETITIONS PER SET**							
4	0.77	0.44	−0.10–1.63	0.083	0.00	0	**14.63 (0.012)**
5	2.17	0.56	1.06–3.27	**<0.001**	0.0	0	
6	0.07	0.40	−0.72–0.86	0.855	0.00	0	
10	−0.04	0.50	−1.02–0.93	0.928	0.00	1	
11	1.28	0.45	0.40–2.16	**0.004**	79.01	0	
12	1.96	0.95	0.09–3.83	0.040	0.0	1	

*CI, confidence interval; I^2^, index of heterogeneity; df, degrees of freedom; SD, standard deviation; SMD, standardized mean differences; p significance level*,

* *p < 0.05*,

** *p < 0.01. Bold values indicate statistically significant values*.

#### Expertise level

Our subgroup analyses indicated that ST produced slightly larger SMDwm magnitudes on CMJ height in trained individuals (SMDwm = 0.81; 95% CI 0.38–1.25; *p* < 0.001; *df* = 10; *I*^2^ = 60.23%) compared with recreationally trained individuals (SMDwm = 0.36; 95% CI 0.01–0.72; *p* = 0.04; *df* = 4; *I*^2^ = 0%). However, the analysis failed to reach the level of significance (*Q* = 3.31, *p* = 0.06).

#### Sex

There was a statistically significant effect of the moderator variable “sex” (males vs. combined males and females) on CMJ height (*Q* = 4.56, *p* = 0.03) but not on SJ height (*Q* = 2.55, *p* = 0.11). ST induced larger effects on CMJ height in males (SMDwm = 0.79; 95% CI 0.42–1.15; *p* < 0.001; *df* = 13; *I*^2^ = 54.74%) compared with the combined males and females group (SMDwm = 0.18; 95% CI −0.24 to 0.60; *p* = 0.40; *df* = 2; *I*^2^ = 0%). A tendency (*p* = 0.11) toward larger ST effects was found for SJ height in males (SMDwm = 0.89; 95% CI 0.27–1.51; *p* = 0.005; *df* = 9; *I*^2^ = 71.97%) compared with the combined group (SMDwm = 0.07; 95% CI −0.72 to 0.86; *p* = 0.85; *df* = 0; *I*^2^ = 0%). The between-group difference did not reach the level of significance.

### Dose-response relationships of strength training on proxies of muscle power

To improve the generalizability and external validity of our study findings, we combined the results from 15 studies that examined the effects of ST on proxies of lower-limb muscle power (CMJ/SJ height) in youth. Such pooling of data was done to explore the effects of continuous training variables on muscle power using meta-regression (Table 4). Besides meta-regression, univariate analyses were computed to identify dose-response relationships for single training modalities (Tables 2, 3).

**Table 4:** Meta regression for training variables of different subscales to predict strength training effects on countermovement and squat jump height.

	**Coefficient**	**Standard error**	**95% lower CI**	**95% upper CI**	***Z* value**	***p* value**
**COUNTERMOVEMENT JUMP**						
Training duration	0.100	0.075	−0.012	0.211	1.754	0.079
Weekly training frequency	−0.105	0.342	−0.775	0.566	−0.306	0.759
Number of exercises	−0.006	0.048	−0.089	0.100	0.118	0.906
Number of sets	0.030	0.195	−0.352	0.412	0.155	0.876
Number of repetitions	0.020	0.069	−0.115	0.155	0.288	0.773
**SQUAT JUMP**						
Training duration	0.214	0.095	0.027	0.401	2.247	**0.025**
Weekly training frequency	0.536	0.972	−1.369	2.440	0.551	0.582
Number of exercises	0.122	0.105	−0.084	0.328	1.159	0.246
Number of sets	−0.251	0.457	−1.146	0.644	−0.549	0.583
Number of repetitions	0.044	0.126	−0.202	0.290	0.349	0.727

*Bold values indicate statistically significant values*.

### Findings from meta-regression

Table 4 shows the results of the meta-regression for the training modalities training duration, weekly training frequency, number of exercises per training session, number of sets, and number of repetitions per training session. The analysis revealed that the modality “training duration” predicted (*p* = 0.02) the effects of ST on SJ height, with longer training durations (>8 weeks) showing larger improvements. For CMJ height, no such training modality was identified (training duration: *p* = 0.07; weekly training frequency: *p* = 0.75; number of exercises: *p* = 0.90; number of sets: *p* = 0.87; number of repetitions: *p* = 0.77).

### Findings from univariate analyses

#### Training duration

There were no significant differences between the observed training period (i.e., ≤ 8 weeks, >8 weeks) for measures of CMJ height (*Q* = 0.05, *p* = 0.82) and SJ height (*Q* = 0.45, *p* = 0.50) (Tables 2, 3).

#### Weekly training frequency

There were no significant differences between the observed weekly training frequencies (i.e., 2, 3 per week) for measures of CMJ height (*Q* = 0.18, *p* = 0.67) and SJ height (*Q* = 0.96, *p* = 0.32) (Tables 2, 3).

#### Number of sets and repetitions

There were no significant differences between the observed number of sets and repetitions for measures of CMJ height (*Q* = 0.78, *p* = 0.85; *Q* = 8.31, *p* = 0.14, respectively) (Table 2).

There was a significant difference with regard to the effects of ST on SJ height for number of repetitions per set (*Q* = 14.63, *p* = 0.01) but not for number of sets per exercise (*Q* = 0.73, *p* = 0.69). More specifically, for number of repetitions, the largest effect with a SMDwm of 2.17 was found for FIVE repetitions to improve SJ height (Tables 3, 4).

#### Training intensity

There were no significant differences between the observed training intensities for measures of CMJ height *Q* = 3.28, *p* = 0.35) and SJ height (*Q* = 1.02, *p* = 0.60) (Tables 2, 3).

### Evaluation of potential risk of bias

Figures 3, 5 show symmetric funnel plots which indicates the absence of publication bias in studies assessing the effects of ST on CMJ and SJ height.

## Discussion

To the best of our knowledge, this is the first meta-analysis to quantify the effects of ST on proxies of muscle power in young athletes and to provide ST-related dose–response relationships for the improvement of muscle power (jump performance). The present meta-analysis found small to moderate effects of ST on CMJ and SJ height. Meta-regression showed that training duration predicted the effects of ST on SJ height in young athletes with longer training durations (>8 weeks) showing larger improvements. This information can help fitness professionals to prescribe the appropriate ST dosage designed to address the specific needs and/or goals of their athletes.

### Effects of strength training on proxies of muscle power

Our results demonstrated that the different forms of ST with various intensities have a greater potential to improve CMJ and SJ heights compared with the active control group. These findings are in line with the results of previous meta-analyses which examined the effects of ST on muscle power (Behm et al., 2017). In fact, Behm and colleagues reported a small effect of ST on jump performance (SMD = 0.52). Lesinski et al. (2016) showed that ST increased muscular power (moderate SMD = 0.80) in young athletes. Moreover, Behringer et al. (2011) and Harries et al. (2012) found similar effects of ST on proxies of muscular power in children and adolescent athletes. However, the novel aspect of the present meta-analysis is that we have examined ST related dose-response relationships for proxies of muscle power (i.e., vertical jump height) in young athletes, which will be discussed below.

From a physiological point of view, preliminary evidence from cross-sectional and longitudinal studies indicate that training induced changes in motor performance strongly rely on neural, muscular, and tendinous adaptations (Legerlotz et al., 2016). Furthermore, the observed small to moderate effects of ST on measures of muscle power in our meta-analysis could be explained by the few included studies that examined proxies of muscular power or the lack of training specificity [an effective transfer of training adaptations occurs when training mimicks the required sport-specific task (Sale and MacDougall, 1981; Behm and Sale, 1993; Behm, 1995)]. In summary, ST can be used as a method to improve proxies of muscular power in child and particularly adolescent athletes. Further research is needed to elucidate the underlying mechanisms following ST in child and adolescent athletes.

### Participant characteristics

Additional sub-analyses indicated no influencing effects of the moderator “expertise level” on proxies of muscle power (i.e., CMJ and SJ height). Only “age” and “sex” influenced ST effects on SJ and CMJ height, respectively. This can most likely be attributed to biological maturation (i.e., children vs. adolescents) and maturational differences (i.e., boys vs. girls), particularly in around and post PHV youth (Behm et al., 2017). In contrast, Behm et al. (2017) reported that untrained youth produced greater ST gains with jump measure than trained youth. This discrepancy in findings could be due to methodological reasons. While we focused on proxies of muscle power only (i.e., CMJ and SJ height), Behm et al. (2017) considered other power parameters (i.e., force and velocity). The results of the current study and previous meta-analyses (Lesinski et al., 2016; Behm et al., 2017) can be explained by age-specific physiological characteristics, particularly the distribution of skeletal muscle mass and fiber type, which are important prerequisites for the generation of muscular power. Further studies are needed to determine the underlying neuromuscular adaptations/mechanism following. Finally, training-induced adaptations have to be separated from growth and maturation.

De Ste Croix et al. (2002) assessed muscle cross-sectional are using magnetic resonance imaging and showed increases in muscle size with age (i.e., from early childhood to late adolescence) that is more pronounced in boys than girls. Besides positive changes in muscle mass evoked by maturation, there is knowledge of sex-specific fiber type growth and its distribution particularly during adolescence. While percentage of type I fibers is equally distributed in boys and girls during childhood, the apparent differences seems to occur during adolescence with females having a lower percentage rates of type I fibers compared with males. In addition, males' type II fibers are larger than their type I fibers which is not the case for females (Vogler and Bove, 1985; Glenmark et al., 1992).

### Dose-response relationships of strength training for muscle power development

#### Training modalities (duration, frequency, number of sets, number of repetitions)

For SJ height, our meta-regression indicated that >8 weeks of training leads to greater training-related effects on SJ height compared with interventions that lasted ≤ 8 weeks (Table 5). For CMJ height, the meta-regression did not identify any parameter to influence ST related effects. This difference in findings for SJ and CMJ height could be due to the biomechanical and physiological differences between CMJs performed in the slow stretch-shortening cycle and SJs performed without the stretch shortening cycle and in concentric mode only (Pupo et al., 2012). Accordingly, Christou et al. (2006) reported significantly larger increases in SJ performance following ST with longer training duration (16 weeks) compared with shorter training duration (8 weeks). With reference to the findings from the literature and our own results, we argue that ST primarily affects SJ performance because ST affords exercises in slow concentric and eccentric mode without using the stretch shortening cycle. Thus, the principle of training specificity could be responsible why ST resulted in larger SJ compared with CMJ improvements.

**Table 5:** Dose–response relationships of strength training to increase muscle power.

**Training modalities**	**Results/most effective dose**	
	**CMJ**	**SJ**
Intensity	Moderate to high	Moderate
Training duration (weeks)	>8 weeks	>8 weeks
Weekly training frequency (sessions per week)	2	3
Number of sets	3	3
Number of repetitions	5^#^	5^*^^#^

* *This moderator significantly influenced ST-related effects on SJ height*,

# *only one study*.

Our analyses revealed no statistically significant effects of the training modalities “weekly training frequency,” “number of sets per exercise,” and “number of repetitions per set” on gains in CMJ height. The calculated SMD data clearly demonstrated small to moderate ST related effects of two to three sessions per week on CMJ height (0.58–0.71) and SJ height (0.74–1.28). Due to the fact that these parameters might be interrelated (e.g., number of sets and number of repetitions), and/or they are heterogeneous across the analyzed studies (e.g., number of training sessions per week), the absence of significance appears plausible. Therefore, further research regarding the influence of these moderating variables is needed. Of note, data presented at the 2012 European College of Sports Science conference showed preliminary evidence that elite powerlifters experienced greater muscular adaptations when total training volume was partitioned over six vs. three weekly training sessions for 15 weeks (Raastad et al., 2012). Future studies should therefore examine whether an additional training related effect is observable if ST is conducted with more than three weekly training sessions.

Concerning the “number of sets per exercise” and “number of repetitions per set,” the largest effects in SJ height gains occurred when children and adolescent athletes used specific number of repetitions (5, 11, and 12 repetitions). Accordingly, Ronnestad et al. (2008) reported that three to five sets and four to six repetitions per set (mean SMDwm = 2.17) during 7 weeks of ST leads to greater effects on SJ height. In addition, two to three sets (mean SMDwm = 1.66) and eight to fifteen repetitions (mean SMDwm = 1.96) and with moderate to high intensity (55–80% of 1RM) leads to greater effects of ST on SJ (Christou et al., 2006). The typical ST protocol for children involves training 2–3 times per week (Malina, 2006), with moderate loads (e.g., 50–60% of 1RM) and higher repetitions (e.g., 15–20 reps) (Faigenbaum et al., 1996, 2009; Lillegard et al., 1997; Christou et al., 2006; Faigenbaum, 2006; Benson et al., 2007; Behm et al., 2008). Lesinski et al. (2016) conducted a meta-analysis with young athletes and computed dose-response relationships following ST for measures of muscle strength in adolescent athletes. Training modalities were calculated in univariate analysis as single factors which is why the results have to be interpreted with caution. The authors observed that a training duration of more than 23 weeks, five sets per exercise, 6–8 repetitions per set, a training intensity of 80–89% of the 1 RM, and 3–4 min rest between sets were most effective single modalities to improve measures of muscle strength (e.g., 1RM) in young athletes.

Concerning the modality “training intensity,” our meta-analysis revealed that this variable did not predict ST-related gains on proxies of muscle power. In contrast, Lesinski et al. (2016) reported that high-intensity ST (i.e., 80–89% of 1RM) was most beneficial to improve muscle strength in young athletes compared with lower training intensities (i.e., 30–39, 40–49, 50–59, 60–69, 70–79% of the 1 RM). Again, this could be explained with the principle of training specificity. Lesinski et al. (2016) examined effects of ST on measures of muscle strength. Thus, intensity during training appears to be an important factor that impacts on gains in muscle strength. We assessed transfer effects of ST on proxies of muscle power. Therefore, training intensity may not play a crucial role as an influencing factor in our analyses. However, it could also be argued that the 1RM is not an adequate tool to assess training intensity, specifically if the goal is to improve muscle power. Rating of perceived exertion (RPE) could be a suitable alternative and surrogate measure for the assessment of training intensity during ST. Accordingly, the guidelines of the American College of Sports Medicine (ACSM) and the American Heart Association (AHA) recommended quantifying internal training load using RPE to set the intensity of ST in both young and older adults (Pollock et al., 2000; Williams et al., 2007). Furthermore, coaches could monitor ST with the use of RPE, movement velocity during resistance exercises (González-Badillo et al., 2015; Pareja-Blanco et al., 2017), or the evaluation of the force-velocity profile (Jiménez-Reyes et al., 2017). Future studies are needed to validate these methods as a training modality during ST in young athletes.

### Study limitations

Our study has several limitations that warrant discussion. First, we computed meta-regression and univariate analyses to identify effective dose-response relationships. While meta-regression controls for other training modalities, univariate analyses do not. Therefore, findings from univariate analyses have to be interpreted with caution. Second, we identified low to moderate heterogeneity between the included studies which could have affected our study outcomes. Third, due to a limited number of studies examining the effects of ST in female young athletes, we were not able to extract findings for females only. Another limitation is that we did not control our quantitative synthesis for variables such as participants' biological maturation and familiarization with jump exercises, and the specific kinetics and kinematics of jump exercises (CMJ vs. SJ). These factors could have influenced training induced adaptations. However, the included studies did not report these information which is why we were not able to adjust for these factors in our analyses. Fourth, due to a lack of data reported in the included studies, we were not able to control for movement velocity and the type of muscle action predominantly performed as well as the specific muscles that were targeted (e.g., upper- or lower-body) during exercise (Gentil et al., 2017).

## Conclusions

In summary, ST is an effective training regime to improve proxies of muscle power in young athletes. Of note, our sub-analyses did not reveal any significant effects of the moderating variable “expertise level” on ST-related outcomes. However, “age” and “sex” moderated ST effects on SJ and CMJ height, respectively. This finding can be explained by maturational and sex-specific physiological characteristics. Findings from the meta-regression showed that longer ST durations (≥8 weeks) are more effective to induce gains in SJ height in both, child and adolescent athletes compared with short-term interventions (<8 weeks). This meta-analysis further detected that training modalities such as training intensity, training frequency, number of sets did not have an impact on ST-related effects on lower-limb muscle power (SJ and CMJ height) in young athletes. As a consequence, there are other not yet identified training modalities that could influence ST-related effects on proxies of muscle power in young athletes. The assessment of RPE, movement velocity or force-velocity profile as monitoring tools for lower-limb muscle power development during ST in young athletes could be promising candidates.

## Author contributions

UG, MS, and AP contributed to the design, analysis, and writing of the manuscript.

### Conflict of interest statement

The authors declare that the research was conducted in the absence of any commercial or financial relationships that could be construed as a potential conflict of interest.
